# Educational performance between the human resource's theoretical paradigm and the practical mentality. Analysis at the European level

**DOI:** 10.3389/fpsyg.2022.1038868

**Published:** 2022-12-06

**Authors:** Romeo Victor Ionescu, Monica Laura Zlati, Valentin Marian Antohi, Nicoleta Cristache, Monica Raducan

**Affiliations:** ^1^Department of Administrative Sciences and Regional Studies, Dunarea de Jos University of Galati, Galaţi, Romania; ^2^Department of Business Administration, Dunarea de Jos University of Galati, Galaţi, Romania; ^3^Department of Finance, Accounting and Economic Theory, Transilvania University of Braşov, Braşov, Romania

**Keywords:** performance in education, human resource, education system, professional motivation, statistical modeling procedures

## Abstract

The issue of performance in education is always topical because it plays an important role in creating the most valuable resource: labor. It has been treated both from a scientific and practical point of view through numerous strategies and optimization techniques, being integrated with the strategies for educational development as a training system at a global level. This research aimed to identify the need for performance and assessed the theoretical and practical dimensions of the European gap in performance in education, given the impact of the global crisis. The analysis was based on a structured questionnaire that is part of a pilot research in which 130 teachers and school managers from Romania and 97 teachers and school managers from five other European countries participated. The results of the study will allow the identification of the human resource's theoretical paradigm and the practical mentality in the education system to improve performance, which the authors propose to use to identify concrete applicable measures in the short term.

## Introduction

There is a particular concern at the level of European decision-makers regarding the continuous improvement of educational performance.

Although differing in specifics, most educational strategies emphasize on some elements such as improvement in curricular experience, the attractiveness of curricular content, improvement in teaching-learning-assessment methods (including digitalization), and introduction of new approaches in accordance with the global objectives regarding sustainability.

In Europe, a strategic framework for European education was developed (European Commission, 2021) and adopted by the Council of Europe Resolution 2021/C 66/01. The framework is built on the Rome Declaration of March 2017, aiming at actions for an EU where young people receive the best education and training, with guaranteed free access to study and jobs across the continent. The Council concluded that education was essential for building a cohesive and inclusive society. In 2017, for the first time, education and training were placed at the center of the European political agenda following which the European Commission established the European Education Area focused on future-oriented education and training systems. The European policy framework was complemented by the annual Sustainable Growth Strategy 2021, which guarantees equal opportunities and inclusive education. Thus, for the 2030 horizon, the achievement and development of the European Education Area will constitute the overall policy objective of the Strategic Framework for European Cooperation in Education and Training as the main instrument to support and implement strategic actions in priority areas. In the short term, until 2025, the aim is to support education and training systems in the EU Member States to ensure the personal, social and professional wellbeing of all citizens by promoting democratic values, equality, social cohesion, active citizenship, and intercultural dialogue.

The other direction for 2025 is to ensure sustainable economic prosperity, green and digital transition, and employability.

For countries outside the EU, such as the UK, achievements in education are correlated with performance indicators in which the score is calculated based on the performance of the educational unit and the Situational Judgement Test (SJT), (UK Foundation Programme, 2020).

These approaches were more intensively reviewed during the pandemic, which involved redesigning the teaching methods and techniques and adapting them to the digital environment, entailing infrastructure, human resource training, and familiarization of the school population with these methods. For the human resource in education, the concept of the online school had a greater impact on the seniority in the profession, because it involved changing traditional methods with new ones and reconfiguring communication practices and obtaining feedback in education.

For these reasons, we witnessed a change in the educational paradigm in recent years that affects the performance of human resources in education both through the proposed objectives in accordance with the paradigm shift and by changing the requirements for alignment with these objectives by human resources currently engaged in the process of education. These elements allow the objective determination of problems of the human resources management's optimization and efficiency in the educational system, the result of which can be determined using statistical modeling procedures by modeling strategies concerning the performance in education.

In this context, we appreciate that the scientific approach comes to cover an area of interest and topicality in the sense of identity based on a statistical survey (questionnaire applied at the level of six European states: United Kingdom, Spain, Greece, Turkey, Romania, and Cyprus) between 12.02.2021 and 28.04.2021, which covered 130 teachers and school managers in Romania and 97 teachers and school managers in other European countries.

The research aimed to identify the need for performance and evaluate the theoretical and practical dimensions of the European gap in performance in education.

The ***research objectives*** are as follows:

**O1:** Analysis of the impact of the current global crisis on performance in education.

**O2:** Performance evaluation based on the outputs generated by the interpretation of the questionnaire.

**O3:** Prospective analysis of vulnerabilities at the level of modifying the management strategy for obtaining performance.

**O4:** Correcting vulnerabilities by adopting viable performance management tools. Comparative analysis by European countries.

Depending on these objectives, the research aimed to identify viable solutions with impact to improve performance in the education system in the short and medium term.

## Literature review

Management related to the increase in educational performance is a topical and extremely interesting research subject. The function of human resources to add value to the educational process justifies the perpetual topicality of the research subject. The dynamics of this process are highly correlated to the dynamics of curricular strategies in education and the emphasis on new education as directives modifying the current performance quantified in the educational process.

**Figure 1:**
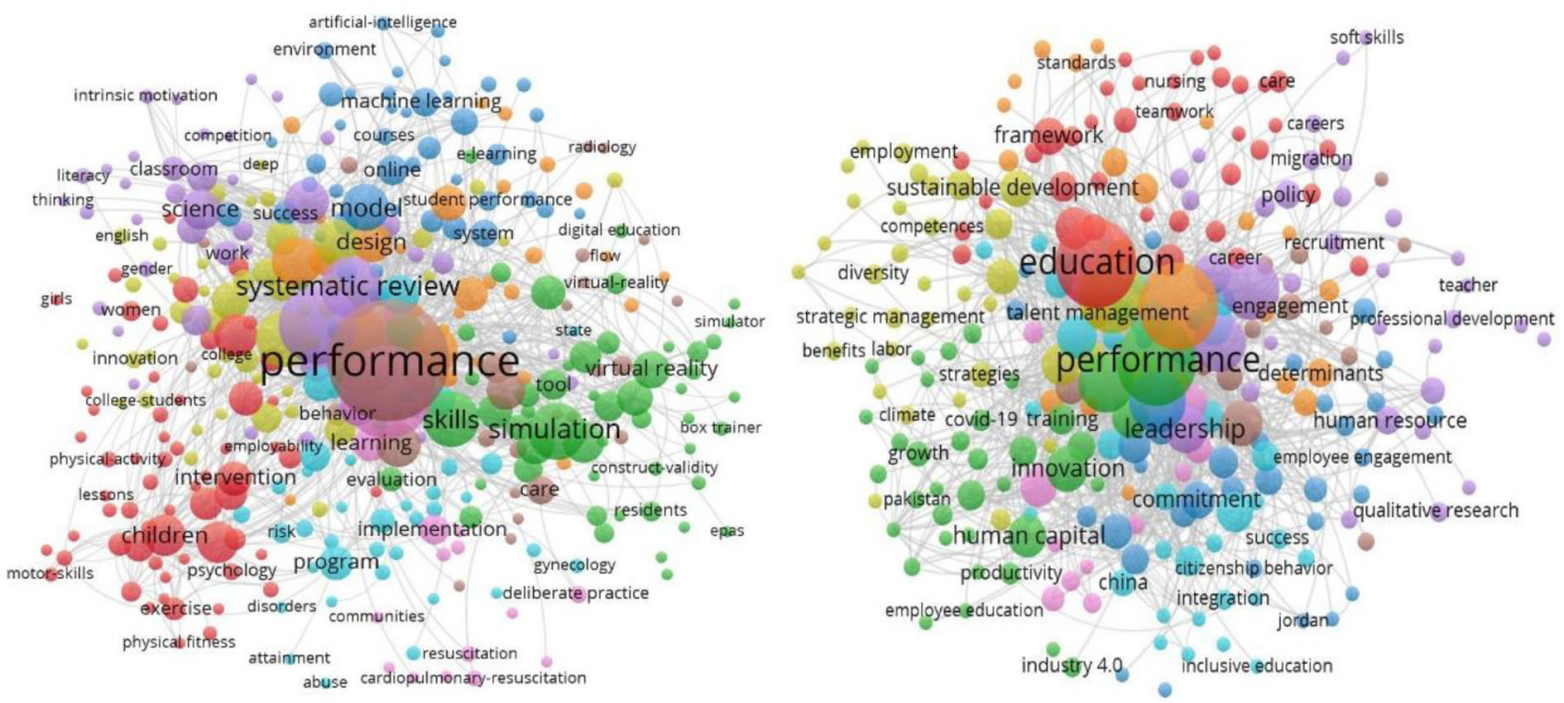
The dimension of the research in the field of education and human resources.

As a summary of the papers presented in the field of education and human resources management (HRM) strategy shows, the authors have recently addressed trends regarding theoretical frameworks and empirical findings of studies (Jiang and Messersmith, 2018). This synthesis leads to the conclusion that approximately half of the works aiming at the strategic management of human resources refer to education and professional training (see Figure 1).

Figure 1 is based on a web of science platform search of 3,279 articles from 2018 to 2022, bibliometrically collected in common databases and designed as a bibliographic data network clustered by keyword groups using Visualizing Scientific Landscape (VosViewer) version 1.6.15, 2020.

From the study of the 1,213 selected publications between the period 2020–2022 on educational performance, it appears that the field is of great interest to researchers; it has a citation rate of 2.76 articles/published work and a Hirsch index of 20 points.

The main connections of performance in education according to the accumulated citations per item included teachers' interpersonal behavior in the communication process, educational applications, deepening digital education, implementation of flexible curricula, the connection of education with artificial intelligence, and the Internet of Things (IoT) applications.

Regarding the effectiveness of human resource management in education, 2,067 articles were selected from the period 2018–2022, showing a double interest in performance in education, the index of citations per item being 4.79 compared to 2.76, and with a Hirsch index of 37 points.

A synthesis of the literature covering 130 papers in the field of quality management in higher education institutions between 1977 and 2015 showed a focus on introducing quality management into the institutions' global management systems. The analysis was undertaken at three levels: a process level, an organizational level, and a quality management principles level (Manatos et al., 2017).

The Workflow of Performance Management developed by Schleicher et al. (2018) integrates performance through the formal and informal components of the individual management process, combining elements of expectation, observation, evaluation, documentation, and feedback to achieve a performance management framework based on the analyzed person's individual and social component taxonomy.

An interesting paper, based on a sample of 129 companies, analyzed the relationship between performance through innovation and knowledge acquisition, and found that knowledge acquisition was positively related to innovation performance; the higher the employee retention, the higher the effects of knowledge acquisition on innovation performance; and the higher the use of HRM practices, the higher the effects of knowledge acquisition on innovation performance. It turns out that the authors anticipated the value of a friendly organizational environment, emphasizing the formative side generating innovation and ultimately performance (Papa et al., 2020).

In the same register of motivational models for increasing human resource performance, other authors propose a model for evaluating HRM performance based on creative processes at the group level and individual proactivity based on trust in management, responsibility for change, and self-motivation, which promotes organization innovation and performance. This study is applicable in education at least in terms of organizing the implementation of good practices and contributing to improving the results of the teaching-learning process by increasing the quality of the educational activities at the level of teacher groups (Lee et al., 2019).

The analysis of the specialized literature resulted in a strong argument in favor of our scientific approach, highlighting the impact of individual experience in managing the activities carried out by teachers in school and outside it through the contribution of responsibility and self-motivation, which generates creativity, a fundamental aspect of teaching.

Thus, we predict,

***Hypothesis 1:*** *Performance in education is the result of combining school activity with extracurricular activities if and only if the teacher's perception of the organization of extracurricular activity is directed to activities with concrete results and a positive impact on performance*.

In obtaining performance, the specialized literature identified a major deceleration factor namely, professional stress. In the teaching profession, this stress is accompanied by interpersonal stress and financial stress, affecting at first the motivation function, and in the final stage, the performance in education. The authors describe motivation as a force multiplier between acceptance, instrumentality, and valence, based on the 1,964 Vroom model (Foy et al., 2019).

Thus, we consider motivation as a major factor in determining performance, which will be introduced as a model variable along with other variables that describe elements of the educational process and aspects of human resource management in education. An extrapolation model of the performance at the level of the management system elaborated by Sales (2019) aims at separating the elements related to the organization's culture (the educational system) from the elements of strategy and planning. These factors can be recomposed into performance keys measurable at the system level through performance targets with the result generating a “reward system”. According to the authors, the study focuses on formal performance. They say it could be complemented by motivational alternatives that coordinate employee behavior.

Some of the organizational culture's elements in their relationship with human resources and performance are presented by Chandler (2018), which shows that HRM activities can influence organizational culture. Thus, the authors consider the organizational culture's elements or the functional elements of the human resources as inputs (attracting through the recruitment process, training, remuneration, and opportunities offered in careers) to build viable outputs (satisfaction, motivation, performance, affiliation to organizational identity). These outputs directly generate an increase in human resources efficiency by reducing absenteeism, improving productivity and quality of work, and adhering to the organizational compliance strategy.

In another approach, Iqbal et al. (2019) studied the influence of the managerial culture on organizational performance through managerial processes segregated by the surplus value achieved by the use of the intellectual capital on the operational branch (up to 80%) and on the innovative branch (up to 85%), both contributing in different percentages to obtain performance in the organization. The proposed model is relevant even if the authors stated the limits of the study based on the small sample size and their resilience only in university research centers.

Adeinat and Abdulfatah propose an interesting approach that calls into question the connection between organizational culture and implementing a successful knowledge management strategy. The study highlights the organizational culture with control functions and divergence of flexibility so that members of the organization can not only benefit from internal advantages focused on cooperation, teamwork, elements of organizational structure, procedures, etc., but can also contribute to the organizational growth through innovation or increased performance and productivity. In this register, the organizing culture creates, disseminates, changes, and applies to the human resource new values through which added value can be obtained in terms of the experience exchange and personal development. The ultimate goal of this approach was to improve the ways it stored the knowledge created by its faculty members and staff (Adeinat and Abdulfatah, 2019).

Another paper addresses organizational planning activities by motivating the leadership of organizational culture to obtain an algorithmic output on employee performance. The analysis is based on two distinct components: motivation and transformational leadership which, through organizational culture, support employee performance (Virgiawan et al., 2021).

The analysis of the specialized literature is a strong argument in favor of our scientific approach, highlighting the impact of organizational comfort as a stimulus of creativity and motivation that generates performance.

Thus, we predict,

***Hypothesis 2:*** *Teachers can be motivated by HRM actions/strategies aimed mainly at promoting a fair status of teachers, attracting the local community and educational partners in collaboration with teachers*.

According to Fleaca et al. (2018), HRM is one of the three main processes that ensure the cross-functional structure of higher education institutions processes. The management function is divided into categories such as value chain planning, monitoring and control of operations carried out through these value chains, monitoring and control of support operations, and quality improvement. The teacher, as an element of human resources, is found at the core of each category, reacting directly with the other structural elements (education, support facilities, and curricular methodologies) that together contribute to the educational process. According to the authors, human resources development is part of another category called the support and administrative process group.

Other researchers attempted to broaden and deepen the empirical focus, theory, and methods used in strategic human resources research, where some have developed an original, integrative framework for theorizing how different combinations of institutions affect management practice, including in education (Doellgast and Marsden, 2019).

The analysis of the specialized literature provides a strong argument in favor of our scientific approach, highlighting the impact of the measures taken at the level of human resources management as an element of stability, reduction of bureaucratic pressure, and creation of premises for performance.

Thus, we predict,

***Hypothesis 3:*** *Increasing flexibility and creating adequate tools to facilitate bureaucratic tasks will have a direct effect on the teacher's performance*.

A mixed-method perspective on the investigation of determinants of effectiveness in quality assurance at higher education institutions has been applied to such institutions in Germany. Based on the collected data, a regression model was developed to determine the quality perception of the managers' position with the need for effective quality assurance. The model identified a causal condition between the need for cooperation between institutions and the increase in the quality of the managerial act, emphasizing that, through the quality function of managers, increased efficiency is obtained and demonstrated by the correlation with perceived effectiveness (Seyfried and Pohlenz, 2018).

Brown et al. (2019) analyzed performance as a relationship between the organization's need for performance, employees' understanding of performance, and the measurable dimension of performance concerning the material dimension of achievement. The meta-analysis of 37 articles showed that the balance between objectives and results was often disturbed by the expectations of the organization and the ability of employees to achieve the proposed objectives.

Under another approach, performance management was considered an element of facilitating the human performance, which could lead to unpleasant experiences for employees in education sometimes. The study was based on responses from 458 teachers and found that there was an indirect relationship between perceived performance management process strength and teacher performance (Waeyenberg et al., 2020).

According to Wang et al. (2018), the performance system of managers in higher education institutions was influenced by factors impacting performance such as the role of stakeholders, academic management through operational and monitoring components, and performance management through the components of establishment, implementation, and evaluation. The limitation of the study, as declared by the authors, included delimiting the study to the academic environment in China represented by a special academic culture, the scale of examination of managerial performance, and the introduction of research only for the higher managerial level, omitting middle-management.

The analysis of this literature provided a strong argument in favor of our scientific approach, highlighting the impact of the managers' interest at all levels in schools, thus ensuring a relationship of stability and organizational growth as prerequisites for performance.

Thus, we predict,

***Hypothesis 4:*** *The interest of high-level management in providing staff motivation tools is one of the desirable strategies with an impact on teachers*. The definition of this working hypothesis is also based on studies carried out in this context by some authors (Barenthien et al., 2020; Holzberger et al., 2021; Zapata-Cardona and Martínez-Castro, 2021; He et al., 2022; Ma, 2022) who correlated educational performance with motivational factors from a deep and correlative scientific perspective.

The lack of research involving cross-country comparisons of the performance of education systems has been highlighted by Stumbriene et al. (2020) and is a further argument in favor of our present research. The authors propose a composite indicator to summarize the performance of education systems. This indicator resulted from a study of Eurostat and Organization for Economic Co-operation and Development statistical databases. The authors calculated the performance scores, produced a ranking of the countries analyzed, and highlighted the managerial implications of this ranking. Another example of quantifying educational performance was presented by Gewirtz et al. (2021) based on the UK situation following a survey of results showing a certain rigidity in defending a test-driven pedagogical culture and a narrowing of the curriculum.

An interesting analysis by Rodríguez-Hernández et al. (2020) questions the correlation between the general level of socioeconomic development, socioeconomic status, and educational performance. The research is based on an extensive meta-analysis (42 studies in the field) and highlights socioeconomic status and performance in education. Socioeconomic status was quantified through education, occupation, income, home resources, and neighborhood resources, while educational performance was measured in terms of achievement, skills, and persistence. Their study showed a weak positive relationship between these two elements.

The meta-analysis method was also used by Sánchez-Álvarez et al. (2020), who studied the connection between emotional intelligence and educational performance based on three main theoretical models. The meta-analysis covered 3,210 articles and a sample of 19,861 participants and found that there was a significant effect of emotional intelligence on educational performance.

The implementation of educational research and institutional policies with effects on education is analyzed by Nazari-Shirkouhi et al. (2020) through a balanced scorecard approach. In this context, the performance evaluation structure was correlated with an integrated multi-criteria fuzzy decision model. The modeling showed that educational income was recognized as one of the most important indicators of educational performance.

The connection between education, socioeconomic environment, technology, and global developments was explored by Sušanj et al. (2020) as a pressure factor in favor of efficiency, professionalism, and professional development of teaching staff. The analysis covered education in Austria, Croatia, and Finland and was aimed to adapt the management of human resources in education to the specific challenges of education in each country analyzed.

An interesting analysis by Jacqmin and Lefebvre (2021) looked at the impact of fiscal decentralization on educational performance. The results of the analysis support the fact that this fiscal decentralization can be favorable to education systems in research-intensive countries.

Teachers' motivation to integrate professional development into practice was studied by Osman and Warner (2020). The analysis used three samples of teachers, researched in an iterative process of developing and validating a specially defined scale. The scale developed by the authors proved to be a tool to measure teachers' expectations of implementation success, its value, and the perceived costs of implementation.

Teachers' teaching practices promoted reading and students' motivation for reading according to Vansteelandt et al. (2020) required establishing a mixed methods design with repeated measures targeting group vs. individual.

Initial teacher training and in-service professional development can include learning opportunities to acquire science-specific competencies as understood by Barenthien et al. (2020). Knowledge about the effects of learning opportunities is relevant to research, policy, and practice. The authors also stress the need for policymakers and professional development providers to ensure sufficient learning opportunities for all teachers.

The approach to the concept of intelligent education by Wang et al. (2021) explored its connection with leadership and human resource capacities. The analysis was based on a simple probabilistic random survey of 349 education employees. The results of the questionnaires were modeled with two dedicated software packages—SPSS and Smart PLS. The study concluded that leadership and human resource capabilities showed a significant influence on intelligent education. The relationship became even stronger through additional investments such as infrastructural innovation.

Hofmeyr (2021) looked at education as a global process from the perspective of the Japanese experience of promoting global human resources. The author notes the need to promote key attitudes, knowledge, and skills that support the eventual “internationalization” of the educational process and global human resources. The international comparative approach to education is also supported by Bellei and Munoz (2021) who studied the regulatory patterns that structure the way the educational provision is organized. They used three types of regulatory models (the traditional bureaucratic professional model, the quasi-market model inspired by neoliberal thinking, and the state evaluative model linked to the notion of new public management) in the case of Chile's education system resulting in an atomized, privatized, and socioeconomically segregated education and found that it improved access to education and increased learning outcomes.

In response to the challenges posed by the pandemic, the education system has been radically transformed through the implementation of smart education. According to Wilson et al. (2021), a shortcoming of this approach is ignoring the importance of leadership and human resources in managing smart education. Their analysis was based on a questionnaire administered to a simple random sample of education employees. Data modeling was done with SPSS-PROCESS Macro software. The main conclusion of the study was that human resources have an indirect conditional effect on intelligent education through the mediating variable of leadership ability.

The preparation of future teachers was studied by Zapata-Cardona and Martínez-Castro (2021) based on a statistical investigation. They found that future teachers' discourse provided clues suggesting that the participants were in the process of developing statistical knowledge as well as developing awareness as critical citizens. As a continuation of the concern of teachers to approach various information statistically, Zieffler et al. (2021) recommended a focus on data science and computational thinking, which could soon position algorithmic models in the school curriculum. The practical research undertaken by the authors highlighted difficulties in learning trajectories, technological tools, and pedagogical approaches in the context of statistical approaches.

A survey of 649 teacher education graduates in Germany by Holzberger et al. (2021) makes the connection between knowledge, beliefs, self-efficacy, and self-regulation on the one hand and teachers' future professional well-being on the other. Following the analysis, the authors identified three profiles of future teachers: a very knowledgeable and committed profile, a profile with below-average knowledge, and a profile with below-average conviction, motivation, and self-regulation.

Collaboration between teachers as an essential aspect in the implementation of inclusive practices in schools is the subject of an analysis by Paju et al. (2022). The survey afferent to this analysis covers 167 Finnish classroom teachers and highlights that, by combining coordination, cooperation, and reflective communication as modes of collaborative action, the results obtained can significantly influence the quality of teaching.

In the same context, teaching practice shows that teachers' motivation is often aimed at social status and well-being. According to He et al. (2022), future teachers should perform emotional work strategically, which can intrinsically motivate them to be teachers. The same issue of teacher identity is addressed by Ma (2022) about teacher commitment and motivation. The efficiency and effectiveness of educators are mediated by these constructs for school problems as well as teaching activity as a whole.

The above review of literature informs the present study to explore the impact of the teaching staff on the performance of their educational institution in particular, and on the performance of national education in general.

## Methodology

**Figure 2:**
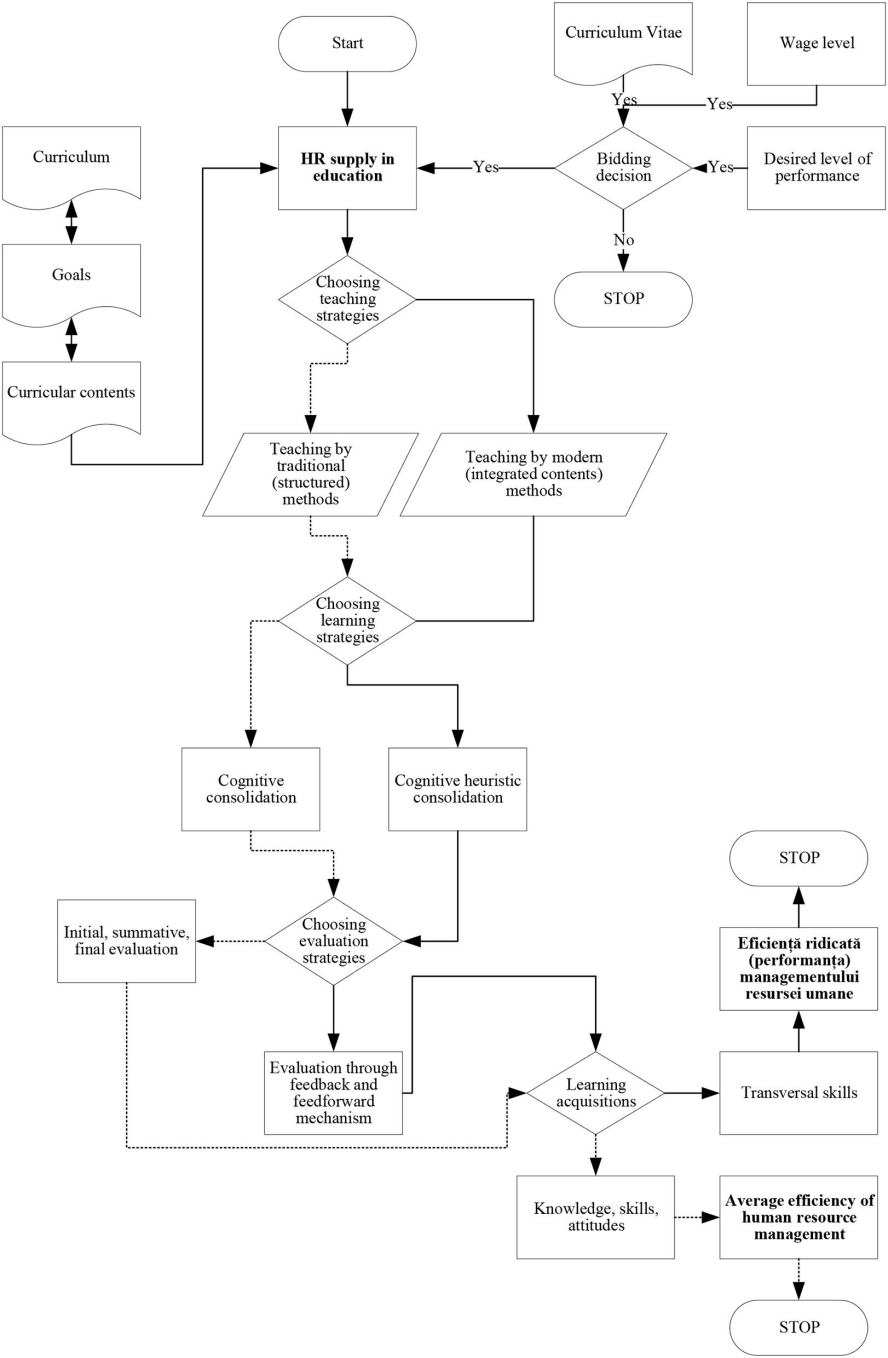
Methodological aspects (logical scheme).

The evaluation methodology was designed differently, based on the teaching-learning strategies, the curricular strategies, and in relation to the proposed performance level. The methodological aspects are illustrated in Figure 2.

**Figure 3:**
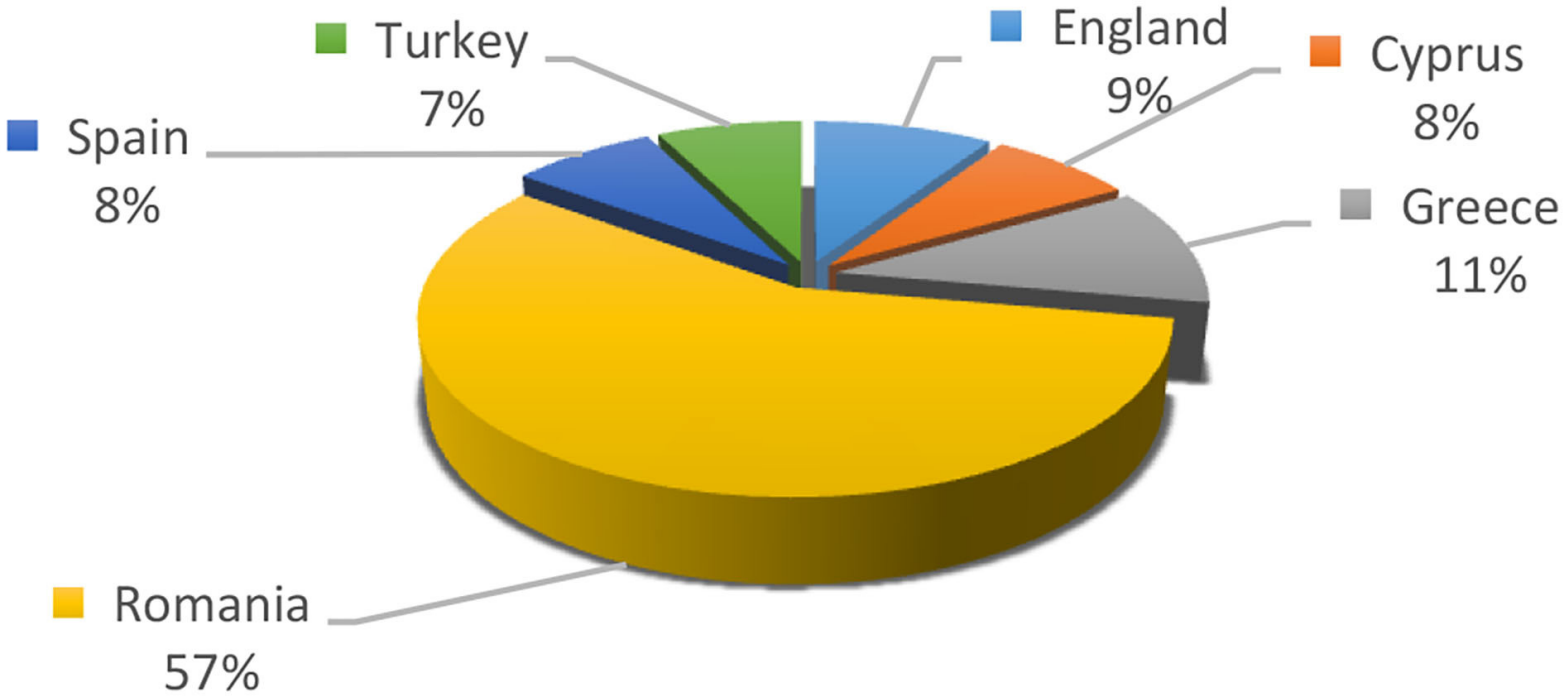
Geographical overview of respondents.

The study used qualitative and quantitative methods of analyzing the answers to the structured questionnaire applied to 227 respondents from six European countries (United Kingdom, Spain, Greece, Turkey, Romania, and Cyprus) between 12.02.2021 and 28.04.2021. The respondents included 21 from the UK, 17 from Cyprus, 25 from Greece, 130 from Romania, 17 from Spain, and 17 from Turkey (Figure 3).

The representativeness of the sample, based on the correlation formula of the statistical total volume of incidence and occupancy of the phenomenon, was calculated according to the formula:


$$\begin{array}{l} n=\frac{t^{2}p\left(1-p\right)N}{t^{2}p\left(1-p\right){+}^{2}\Delta N}\\ =\frac{{\left(1.96\right)}^{2}0.95\left(1-0.95\right)300000}{{\left(1.96\right)}^{2}0.95\left(1-0.95\right)+\left(0.05^{2}\right)300000}=\frac{54742.8}{750.18}\\ =72.97\approx 73\ll 227 \end{array}$$

where: *n*—the volume of the representative sample; *t*—coefficient of the significance test (1.96); *p*—incidence of the phenomenon (0.95); Δ–maximum permissible error (0.05); *N*—a statistical total of teachers in the six EU member states (300,000 teachers and school managers).

The purpose of the questionnaire was to quantify the current degree of dependence of human resource management (in pre-university education institutions) on the factors generating changes in the education system, as well as to identify opportunities for improving management performance based on motivational factors and factors extrinsic to the education process while identifying the main systemic vulnerabilities of human resource management.

The motivational factors targeted by the analysis were salary level, job satisfaction, degree of adaptability to curriculum requirements, additional needs of teachers employed in the system, need for recognition, etc. Extrinsic factors relate to the dynamics of national education systems in relation to the European training strategy in the European public system, changes in education policy and their impact on human resource management, and other conjunctural factors likely to affect human resource management.

- The sample of respondents included:
- new teachers;
- school managers in state and private pre-university education institutions; and
- teachers of various specializations.
  The questionnaire was made up of 24 questions and was structured in two parts. The questions were asked of teachers and school managers in the sample countries, and a table of overall scores was produced for each.
  Data analysis procedures based on frequency statistical series were used, with methods that allowed not only the stratification of vulnerabilities according to the respondent's country of origin but also the identification of general opportunities rooted in the generalized structure of the European public pre-university education system.
  The main objective of the questionnaire was to assess performance and to identify vulnerabilities in achieving the optimal level of performance based on motivational factors and extrinsic factors of the educational process, an aspect that captures both materiality and the theoretical definition of human resources in education as a generator of performance.

The structure of the questionnaire covered:

- identifying the role of human resources management in the performance of school units;
- quantifying the teachers' performance;
- evaluation of the performance output at the level of the school population;
- identifying the role of extracurricular activities in obtaining school performance in pre-university education units;
- quantifying the impact of increasing teachers' motivation.

The motivational factors targeted by the analysis were salary levels, job satisfaction, adaptability degree to the curriculum requirements, the additional needs of teachers employed in the system, the need for recognition, etc.

The extrinsic factors concern the dynamics of the national education systems in relation to the European training strategy in the European public education system, changes in education policy and their repercussions on HRM, and other conjunctural factors likely to affect HRM.

Through modeling, it was proposed to prove the working *hypotheses H1–H4* as established following the study of the literature.

The proposed model was based on the least square's method using multiple linear regression and was developed using IBM—SPSS version 25 statistical programs and Gretl version 2020. The model starts from the principle that the adequacy of human resources policies is based on the interconnection of subjective and objective motivational factors in accordance with the teacher's perception of performance and is a mixed strategy that has been tested according to national and international customs.

Following the statistical modeling, the Enter test method was applied, a method that generated valid values for all regressive variables with the dependent variable “Education performance is the result of combining school activity with extracurricular activities” (EDPERF-EXTRACURR). No variables were excluded after applying the Enter Method. The statistical summary of the model revealed an unadjusted coefficient of determination of over 70%, which confirmed the statistical significance of the model and the fact that it is an optimal model for the analysis of HRM options for teachers' performance (see Table 1).

**Table 1:** Descriptive statistics of the variables subject to modeling.

**Indicators**	**Questions**	**Possible answers**					**Mean**	**Median**	**Std. error of mean**	**Std. deviation**	**Variance**
EDMRU-POLCOER—A coherent educational policy provides the optimal framework for school performance.	In the current educational context, you consider that an efficient human resources management leads to a complex of solutions for the following aspects: [1. Coherent educational policies]	1. Fully agree	2. Agree	3. Neither agree nor disagree	4. Disagree	5. Fully disagree	1.6344	2	0.046	0.687	0.472
EDMRU-ANGPERSCOMP—The employment of competent staff is a prerequisite for the schools' performance.	In the current educational context, you consider that an efficient human resources management leads to a complex of solutions for the following aspects: [2. Hiring competent staff]	1. Fully agree	2. Agree	3. Neither agree nor disagree	4. Disagree	5. Fully disagree	1.6123	1	0.050	0.752	0.566
EDMRU-MENTPERSCOMP—Maintaining valuable employees in the system is a condition for the school units' performance.	In the current educational context, you consider that an efficient human resources management leads to a complex of solutions for the following aspects: [3. Keeping valuable employees in the system]	1. Fully agree	2. Agree	3. Neither agree nor disagree	4. Disagree	5. Fully disagree	1.5815	1	0.049	0.744	0.554
EDMRU-FORMCC—Continuing education of the teachers is a way to maintain the schools' performance.	In the current educational context, you consider that an efficient human resources management leads to a complex of solutions for the following aspects: [4. Continuous teachers' training]	1. Fully agree	2. Agree	3. Neither agree nor disagree	4. Disagree	5. Fully disagree	1.8855	2	0.069	1.041	1.084
EDMRU-ASIGRES—Ensuring the necessary material resources is the logistical support that leads to performance in schools.	In the current educational context, you consider that an efficient human resources management leads to a complex of solutions for the following aspects: [6. Providing the necessary material resources]	1. Fully agree	2. Agree	3. Neither agree nor disagree	4. Disagree	5. Fully disagree	1.7401	2	0.057	0.861	0.742
FLEXMG-PERF—The flexibility of the management team positively influences the performance of the school unit.	The flexibility shown by the management team of the educational unit positively influences the performance of the school institution:	1. Fully agree	2. Agree		3. Neither agree nor disagree		1.6872	2	0.050	0.749	0.561
EDMRU-DESCENTR- Decentralization of the education system negatively influences the schools' performance.	On a scale from 1 to 5, which are the main aspects of human resources management that negatively affect the performance of the school units (1—very low; 5—very high): [1. Decentralization of the education system]	1. Very low	2. Low	3. Medium	4. High	5. Very high	2.9471	3	0.078	1.178	1.387
EDMRU-BIROCR—Bureaucracy has a negative influence on the performance in schools.	On a scale from 1 to 5, which are the main aspects of human resources management that negatively affect the performance of the school units (1—very low; 5—very high): [2. Bureaucracy/ too much paperwork]	1. Very low	2. Low	3. Medium	4. High	5. Very high	4.2555	5	0.063	0.948	0.899
EDMRU-MEDEC—The social and economic environment from which students come negatively affects the level of performance in schools.	On a scale from 1 to 5, which are the main aspects of human resources management that negatively affect the performance of the school units (1—very low; 5—very high): [3. The social and economic environment from which the student comes]	1. Very low	2. Low	3. Medium	4. High	5. Very high	3.5859	4	0.071	1.067	1.137
EDMRU-DEFPREG- Inadequate teacher training affects the performance of the education system.	On a scale from 1 to 5, which are the main aspects of human resources management that negatively affect the performance of the school units (1—very low; 5—very high): [4. Inadequate teacher training]	1. Very low	2. Low	3. Medium	4. High	5. Very high	4.0132	4	0.064	0.957	0.916
EDMRU-MOBILIT—The lack of the internationalization elements negatively conditions the level of the performance in education.	On a scale from 1 to 5, which are the main aspects of human resources management that negatively affect the performance of the school units (1—very low; 5—very high): [5. Lack of internationalization elements (international training projects/courses, mobility)]	1. Very low	2. Low	3. Medium	4. High	5. Very high	3.5551	4	0.068	1.026	1.053
PERFANGJ-ELEV- As direct beneficiaries of the education system, the students are interested in the performance of the employees in education.	Who do you think would be interested in the performance of educational staff? [1. Students]	1. Very low	2. Low	3. Medium	4. High	5. Very high	3.6256	4	0.074	1.111	1.235
PERFANGJ-EDMGSUP- Higher level management in education is directly interested in the level of performance of the employees in this system.	Who do you think would be interested in the performance of educational staff? [2. Higher level management in education]	1. Very low	2. Low	3. Medium	4. High	5. Very high	4.1762	4	0.061	0.919	0.845
PERFANGJ-EDMGINF- Representatives of middle and lower level management in education are concerned about the performance of the education employees.	Who do you think would be interested in the performance of educational staff? [3. Middle and lower level management in education]	1. Very low	2. Low	3. Medium	4. High	5. Very high	4.0352	4	0.065	0.982	0.963
PERFANGJ-SAL—Employees in the education system are interested in performance.	Who do you think would be interested in the performance of educational staff? [4. Employees]	1. Very low	2. Low	3. Medium	4. High	5. Very high	3.837	4	0.061	0.914	0.836
PERFANGJ-PARINTI—Parents of students are concerned about the performance of the education system.	Who do you think would be interested in the performance of educational staff? [5. The parents of the students]	1. Very low	2. Low	3. Medium	4. High	5. Very high	4.0485	4	0.062	0.937	0.878
PERFANGJ-COMLOC—The local community is concerned with the educational performance.	Who do you think would be interested in the performance of educational staff? [6. The local community]	1. Very low	2. Low	3. Medium	4. High	5. Very high	3.489	3	0.073	1.094	1.198
PERFANGJ-ONG—NGO representatives are concerned about performance in education.	Who do you think would be interested in the performance of educational staff? [7. NGOs]	1. Very low	2. Low	3. Medium	4. High	5. Very high	2.9251	3	0.078	1.174	1.379
PERFANGJ-ADM—ATU (Administrative Teritorrial Units) representatives are interested in the performance in the school units.	Who do you think would be interested in the performance of educational staff? [8. Administration/Municipalities]	1. Very low	2. Low	3. Medium	4. High	5. Very high	3.1586	3	0.080	1.202	1.444
OPPERF-DESCENTR—The decentralization of the education system offers the opportunity to increase the teachers' performance.	Identify the extent to which the following aspects represent opportunities that lead to increased teacher performance (1—very low; 5—very high): [1. Decentralization of the education system]	1. Very low	2. Low	3. Medium	4. High	5. Very high	3.4493	4	0.070	1.056	1.116
OPPERF-POLCOER—A system of coherent educational policies can lead to increase the teacher performance.	Identify the extent to which the following aspects represent opportunities that lead to increased teacher performance (1—very low; 5—very high): [2. Consistent and realistic educational policies]	1. Very low	2. Low	3. Medium	4. High	5. Very high	4.3524	5	0.053	0.798	0.636
OPPERF-STATUT—Promoting a fair teacher status can lead to increase the teacher performance.	Identify the extent to which the following aspects represent opportunities that lead to increased teacher performance (1—very low; 5—very high): [3. Promoting a correct status of the teacher]	1. Very low	2. Low	3. Medium	4. High	5. Very high	4.5419	5	0.048	0.724	0.524
OPPERF-RECOMP—The implementation of a correct system of rewarding teachers contributes to increasing their performance.	Identify the extent to which the following aspects represent opportunities that lead to increased teacher performance (1—very low; 5—very high): [4. Reward teachers with great results]	1. Very low	2. Low	3. Medium	4. High	5. Very high	4.2819	4	0.058	0.877	0.770
OPPERF-FORMCC—Access to training courses contributes to increasing the teachers' performance.	Identify the extent to which the following aspects represent opportunities that lead to increased teacher performance (1—very low; 5—very high): [5. Access to free training courses]	1. Very low	2. Low	3. Medium	4. High	5. Very high	4.2291	5	0.065	0.978	0.956
OPPERF-MASSMED—Greater social media visibility on school performance contributes to increasing teacher's performance.	Identify the extent to which the following aspects represent opportunities that lead to increased teacher performance (1—very low; 5—very high): [6. The impact of social media on school performance]	1. Very low	2. Low	3. Medium	4. High	5. Very high	3.9471	4	0.067	1.016	1.033
BENMRU-PERCEPSOC—Improving social perception of the teaching profession will generate high interest in increasing the teachers 'performance.	State to what extent the human resources management generates benefits in the performance of the teachers (1—very low; 5—very high): [1. Improving the social perception of the teaching profession]	1. Very low	2. Low	3. Medium	4. High	5. Very high	4.163	4	0.057	0.865	0.748
BENMRU-COLAB—A good collaboration with stakeholders generates teachers' performance.	State to what extent the human resources management generates benefits in the performance of the teachers (1—very low; 5—very high): [2. Better collaboration with the local community/educational partners]	1. Very low	2. Low	3. Medium	4. High	5. Very high	3.9427	4	0.066	0.987	0.975
BENMRU-IMPLIC—Greater involvement of the teachers in the training process generates educational performance.	State to what extent the human resources management generates benefits in the performance of the teachers (1—very low; 5—very high): [3. Increasing the degree of involvement of the teacher in the teaching process]	1. Very low	2. Low	3. Medium	4. High	5. Very high	4.0352	4	0.069	1.034	1.070
BENMRU-PREDICT—Stability and predictability are factors that motivate performance in education.	State to what extent the human resources management generates benefits in the performance of the teachers (1—very low; 5—very high): [4. Strengthening the feeling of stability and predictability of the educational act]	1. Very low	2. Low	3. Medium	4. High	5. Very high	4.2291	4	0.062	0.927	0.859
BENMRU-COMPETITIE—A fair competitive environment can contribute to the teachers' performance.	State to what extent the human resources management generates benefits in the performance of the teachers (1—very low; 5—very high): [5. Promoting fair competition]	1. Very low	2. Low	3. Medium	4. High	5. Very high	3.9163	4	0.063	0.953	0.909
MOTIV-ECHMRU—A coherent and equitable human resource management promotes increased teacher motivation.	To what extent do you consider that these aspects represent factors that lead to the increase of teachers' motivation for teaching activity (marked with grade 5—very high ... grade 1—very low): [Coherent and equitable human resource management]	1—Very low	2—Low	3—Regardless	4—High	5—Very high	4.1278	4	0.056	0.850	0.723
MOTIV-STABIL—The stable working environment from the legislative and curricular point of view leads to the increase of the teachers' motivation.	To what extent do you consider that these aspects represent factors that lead to the increase of teachers' motivation for teaching activity (marked with grade 5—very high ... grade 1—very low): [Stable working environment from a legislative and curricular point of view]	1—Very low	2—Low	3—Regardless	4—High	5—Very high	3.978	4	0.057	0.864	0.747
MOTIV-CLIMAT—The social climate and the harmonious relationships with colleagues/school manager increase the teachers' motivation.	To what extent do you consider that these aspects represent factors that lead to the increase of teachers' motivation for teaching activity (marked with grade 5—very high ... grade 1—very low): [Social climate and relationships with colleagues/school manager]	1—Very low	2—Low	3—Regardless	4—High	5—Very high	3.8458	4	0.060	0.911	0.830
MOTIV-DOTARE—The material endowment of the educational unit favors the teachers' motivation for the teaching activity.	To what extent do you consider that these aspects represent factors that lead to the increase of teachers' motivation for teaching activity (marked with grade 5—very high ... grade 1—very low): [Material endowment of the educational unit]	1—Very low	2—Low	3—Regardless	4—High	5—Very high	3.6211	4	0.064	0.958	0.918
MOTIV-ORGANIZ—The organization of the work (definition of the tasks, effort, communication, feedback) increases the degree of motivation of the education staff.	To what extent do you consider that these aspects represent factors that lead to the increase of teachers' motivation for teaching activity (marked with grade 5—very high ... grade 1—very low): [Work organization: defining tasks, effort, communication, feedback]	1—Very low	2—Low	3—Regardless	4—High	5—Very high	3.8018	4	0.063	0.955	0.912
MOTIV-SALARIZ—The salary level increases the teachers' degree of motivation.	To what extent do you consider that these aspects represent factors that lead to the increase of teachers' motivation for teaching activity (marked with grade 5—very high ... grade 1—very low): [Employee payment level]	1—Very low	2—Low	3—Regardless	4—High	5—Very high	4.1498	4	0.036	0.544	0.296
MOTIV-STATUT—The positive perception of the teacher's status in the society favors the increase of the teachers' motivation.	To what extent do you consider that these aspects represent factors that lead to the increase of teachers' motivation for teaching activity (marked with grade 5—very high ... grade 1—very low): [Positive perception of the status of the teacher in society]	1—Very low	2—Low	3—Regardless	4—High	5—Very high	3.5066	4	0.067	1.006	1.012
MOTIV-RECOMPENS—Appropriate remuneration for the work performed by teachers (the amount of salary, other financial incentives, recognition/appreciation of work results, promotion prospects) have a positive influence on their motivation.	Appropriate remuneration of the work performed by the teacher (the amount of salary, other financial incentives, recognition/appreciation of work results, promotion prospects) positively influences his motivation.	1. Totally agree	2. Agree		3. Neither agree nor disagree		1.978	2	0.043	0.655	0.429
RESPMOTIV-EDMGSUP—An appropriate degree of motivation among teachers depends on senior management.	Who do you consider to be responsible for maintaining an appropriate degree of motivation of employees in the pre-university education system? [Higher level management in education]	1—Very low	2—Low	3—Regardless	4—High	5—Very high	3.3436	4	0.090	1.352	1.828
RESPMOTIV-EDMGINF—An appropriate degree of motivation among teachers depends on middle and lower level management.	Who do you consider to be responsible for maintaining an appropriate degree of motivation of employees in the pre-university education system? [Intermediate and lower level management in education]	1—Very low	2—Low	3—Regardless	4—High	5—Very high	4.1982	4	0.065	0.982	0.965
RESPMOTIV-SAL—An appropriate degree of motivation among teachers depends on their self-motivation.	Who do you consider to be responsible for maintaining an appropriate degree of motivation of employees in the pre-university education system? [Employees, by self-motivation]	1—Very low	2—Low	3—Regardless	4—High	5—Very high	4.3304	4	0.050	0.747	0.558
RESPMOTIV-PARINTI—An appropriate degree of motivation among teachers depends on the parents of the students.	Who do you consider to be responsible for maintaining an appropriate degree of motivation of employees in the pre-university education system? [Parents of students]	1—Very low	2—Low	3—Regardless	4—High	5—Very high	3.1454	4	0.071	1.077	1.160
RESPMOTIV-COMLOC—An appropriate degree of motivation among teachers depends on the representatives of the local communities.	Who do you consider to be responsible for maintaining an appropriate degree of motivation of employees in the pre-university education system? [Representatives of the local community]	1—Very low	2—Low	3—Regardless	4—High	5—Very high	2.9163	3	0.076	1.151	1.325
RESPMOTIV-ADM—An appropriate degree of motivation among teachers depends on local public administration representatives.	Who do you consider to be responsible for maintaining an appropriate degree of motivation of employees in the pre-university education system? [Representatives of local public administration/town halls]	1—Very low	2—Low	3—Regardless	4—High	5—Very high	2.8767	3	0.080	1.206	1.454
MOTIV-EDPERF—Teachers' motivation is in an interdependent relationship with the school performance's level.	There is a close interdependence between the motivation of teachers and the level of school performance obtained in the pre-university education system.	1. Totally agree	2. Agree		3. Neither agree nor disagree		1.8106	2	0.049	0.731	0.535
PROVOCMRU-TRANSPCE—Lack of transparency in communicating with students can affect students' performance.	To what extent do you consider that the following aspects of the HRM represent challenges for students' performance? [1. Lack of transparency in communication with the Student Council representatives]	1. Low	2. Regardless		3. High		2.2775	2	0.049	0.745	0.555
PROVOCMRU-CONSULTCE—Taking students' feedback is a way to ensure their performance.	To what extent do you consider that the following aspects of the HRM represent challenges for students' performance? [2. Consulting the Student Council in matters of interest to them]	1. Low	2. Regardless		3. High		2.5198	3	0.043	0.647	0.419
PROVOCMRU-LEGIS—An appropriate legislative framework can ensure student-level performance.	To what extent do you consider that the following aspects of the HRM represent challenges for students' performance? [3. Legislative framework]	1. Low	2. Regardless		3. High		2.2599	2	0.047	0.715	0.512
PROVOCMRU-RECOMPENS—Rewarding excellence results in students' performance.	To what extent do you consider that the following aspects of the HRM represent challenges for students' performance? [4. Consistent material rewards of prize-winning students at the Olympiads and school competitions]	1. Low	2. Regardless		3. High		2.4978	3	0.043	0.641	0.410
PROVOCMRU-EXCELENTA—Funding on the format of centers of excellence generates performance among students.	To what extent do you consider that the following aspects of the HRM represent challenges for students' performance? [5. Appropriate financing of students “Excellence Centers”]	1. Low	2. Regardless		3. High		2.3392	2	0.048	0.725	0.526
FIN-EDPERF—An appropriate funding system facilitates the achievement of the school performance.	There is a positive relationship between the funding system and school performance:	1. Fully agree	2. Agree	3. Neither agree nor disagree	4. Disagree	5. Fully disagree	2.0088	2	0.069	1.039	1.080
ANGPERSCOMP-EDPERF—Promoting a system of teacher recruitment at school level contributes to increasing performance in education.	The recruitment of the teaching staff at the level of the educational unit has the effect of increasing school performance level:	1. Fully agree	2. Agree	3. Neither agree nor disagree	4. Disagree	5. Fully disagree	1.9648	2	0.065	0.972	0.946
EDPERF-IMPACT—The design and development of extracurricular activities favors the achievement of performance in education.	Organizing extracurricular activities has a positive impact on performance in education:	1. Fully agree	2. Agree	3. Neither agree nor disagree	4. Disagree	5. Fully disagree	1.7181	2	0.048	0.716	0.513

The shape linear regression function was defined as:


(1) $$\begin{array}{l} F=\overset{n}{\underset{i=1}{\sum }}x_{i}\alpha_{i}+\varepsilon \end{array}$$

This multiple regression function provides the correlation of the dependent variable F with respect to the regressors x_i_, according to the regression coefficients α_i_, with a residual representation error ε of the correlation. We consider the function F validly represented if and only if for any x_i_, i∈[1, *n*] → (∃)α_*i*_≠0 such that the correlation value of the function by the coefficient of determination computed by the least squares method falls in the range [0.6, 1]. For particular cases, such as determining performance in education based on motivational factors and motivation as a determinant of the management policies, the function F was defined as follows:

performance in education based on motivational factors:


(2) $$\begin{array}{l} EDPERF-EXTRACURR=\overset{n}{\underset{i=1}{\sum }}x_{i}\alpha_{i}+\varepsilon \end{array}$$

where: *x*_*i*_ = {RESPMOTIV-EDMGINF, PERFANGJ-ELEV, PROVOCMRU-RECOMPENS, PERFANGJ-EDMGINF, MOTIV-CLIMAT, EDMRU-DESCENTR, EDMRU-ANGPERSCOMP, ANGPERSCOMP-EDPERF, PROVOCMRU-TRANSPCE, EDMRU-MEDEC, MOTIV-ECHMRU, OPPERF-DESCENTR, MOTIV-RECOMPENS, EDPERF-IMPACT, RESPMOTIV-COMLOC, PERFANGJ-ADM, OPPERF-RECOMP, FIN-EDPERF, MOTIV-DOTARE, EDMRU-MOBILIT, PROVOCMRU-LEGIS, RESPMOTIV-SAL, MOTIV-STATUT, OPPERF-MASSMED, PROVOCMRU-CONSULTCE, EDMRU-ASIGRES, PERFANGJ-SAL, EDMRU-BIROCR, BENMRU-PREDICT, FLEXMG-PERF, EDMRU-DEFPREG, PERFANGJ-PARINTI, MOTIV-SALARIZ, BENMRU-PERCEPSOC, PROVOCMRU-EXCELENTA, EDMRU-POLCOER, RESPMOTIV-PARINTI, MOTIV-STABIL, OPPERF-POLCOER, MOTIV-EDPERF, MOTIV-ORGANIZ, OPPERF-FORMCC, BENMRU-COLAB, PERFANGJ-ONG, BENMRU-COMPETITIE, PERFANGJ-EDMGSUP, EDMRU-MENTPERSCOMP, OPPERF-STATUT, RESPMOTIV-EDMGSUP, PERFANGJ-COMLOC, BENMRU-IMPLIC, EDMRU-MOTIV, RESPMOTIV-ADM, EDMRU-FORMCC} (see Table 1).

performance in education based on motivational factors:


(3) $$\begin{array}{l} RESPMOTIV-EDMGSUP=\overset{n}{\underset{i=1}{\sum }}x_{i}\alpha_{i}+\varepsilon \end{array}$$

where: *x*_*i*_ = {OPPERF-FORMCC, MOTIV-DOTARE, PROVOCMRU-CONSULTCE, MOTIV-CLIMAT, FIN-EDPERF, PERFANGJ-ELEV, OPPERF-DESCENTR, RESPMOTIV-ADM, EDMRU-DESCENTR, EDMRU-ANGPERSCOMP, EDMRU-MEDEC, EDPERF-EXTRACURR, MOTIV-ECHMRU, PROVOCMRU-EXCELENTA, MOTIV-RECOMPENS, PROVOCMRU-TRANSPCE, PERFANGJ-ADM, ANGPERSCOMP-EDPERF, PERFANGJ-EDMGINF, RESPMOTIV-SAL, MOTIV-STATUT, OPPERF-MASSMED, EDMRU-MOBILIT, EDMRU-ASIGRES, PROVOCMRU-LEGIS, BENMRU-PREDICT, EDMRU-BIROCR, FLEXMG-PERF, PERFANGJ-PARINTI, PERFANGJ-SAL, EDMRU-DEFPREG, MOTIV-SALARIZ, RESPMOTIV-EDMGINF, BENMRU-PERCEPSOC, RESPMOTIV-PARINTI, EDMRU-POLCOER, PROVOCMRU-RECOMPENS, OPPERF-RECOMP, MOTIV-STABIL, MOTIV-EDPERF, OPPERF-POLCOER, MOTIV-ORGANIZ, BENMRU-COLAB, PERFANGJ-ONG, BENMRU-COMPETITIE, PERFANGJ-EDMGSUP, EDMRU-MENTPERSCOMP, OPPERF-STATUT, EDPERF-IMPACT, BENMRU-IMPLIC, PERFANGJ-COMLOC, EDMRU-MOTIV, EDMRU-FORMCC, RESPMOTIV-COMLOC} (see Table 2).

**Table 2:** Summary of the linear regression model on motivation.

**Model^a^^,^ ^b^**	**R**	**R square**	**Coefficient of determination**	**Standard error of the estimate**	**Change statistics**	
					**R square**	**F**
Motivation	0.856	0.733	0.649	0.449	0.733	8.730
	df1	df2	Sig. F	Durbin-Watson
	54	172	0.000	2.250

a Predictors: (Constant), RESPMOTIV-EDMGINF, PERFANGJ-ELEV, PROVOCMRU-RECOMPENS, PERFANGJ-EDMGINF, MOTIV-CLIMAT, EDMRU-DESCENTR, EDMRU-ANGPERSCOMP, ANGPERSCOMP-EDPERF, PROVOCMRU-TRANSPCE, EDMRU-MEDEC, MOTIV-ECHMRU, OPPERF-DESCENTR, MOTIV-RECOMPENS, EDPERF-IMPACT, RESPMOTIV-COMLOC, PERFANGJ-ADM, OPPERF-RECOMP, FIN-EDPERF, MOTIV-DOTARE, EDMRU-MOBILIT, PROVOCMRU-LEGIS, RESPMOTIV-SAL, MOTIV-STATUT, OPPERF-MASSMED, PROVOCMRU-CONSULTCE, EDMRU-ASIGRES, PERFANGJ-SAL, EDMRU-BIROCR, BENMRU-PREDICT, FLEXMG-PERF, EDMRU-DEFPREG, PERFANGJ-PARINTI, MOTIV-SALARIZ, BENMRU-PERCEPSOC, PROVOCMRU-EXCELENTA, EDMRU-POLCOER, RESPMOTIV-PARINTI, MOTIV-STABIL, OPPERF-POLCOER, MOTIV-EDPERF, MOTIV-ORGANIZ, OPPERF-FORMCC, BENMRU-COLAB, PERFANGJ-ONG, BENMRU-COMPETITIE, PERFANGJ-EDMGSUP, EDMRU-MENTPERSCOMP, OPPERF-STATUT, RESPMOTIV-EDMGSUP, PERFANGJ-COMLOC, BENMRU-IMPLIC, EDMRU-MOTIV, RESPMOTIV-ADM, EDMRU-FORMCC.

b Dependent Variable: EDPERF-EXTRACURR.

The theoretically modeled results based on statistical regression functions were applied at the database level to the observations drawn from the structured questionnaire, resulting in the performance models detailed in the Results section.

## Results

Based on the regression indicators, the econometric model was constructed, using linear regression and the least squares method, the results are presented in Table 2.

The coefficient of determination indicates a statistical representativeness of the model of 73% in the unadjusted version and 65% in the adjusted version, which allows the validation of the model from a statistical point of view. The ANOVA test showed that the proportion of the sum of the squares of the regressors was three times higher than the sum of the residual squares, and the number of degrees of freedom was 54 for the regression variables and 172 for the residual variables. The Sig coefficient was 0, which confirms the homogeneity of the proposed model (see Table 3).

**Table 3:** ANOVA table on motivation.

**Model^a^^,^ ^b^**		**Sum of Squares**	**df**	**Mean Square**	**F**	**Sig**.
Motivation	Regression	95.002	54	1.759	8.730	0.000
	Residual	34.663	172	0.202		
	Total	129.665	226			

a Dependent Variable: EDPERF-EXTRACURR (tabelul 3.3).

b Predictors: (Constant), RESPMOTIV-EDMGINF, PERFANGJ-ELEV, PROVOCMRU-RECOMPENS, PERFANGJ-EDMGINF, MOTIV-CLIMAT, EDMRU-DESCENTR, EDMRU-ANGPERSCOMP, ANGPERSCOMP-EDPERF, PROVOCMRU-TRANSPCE, EDMRU-MEDEC, MOTIV-ECHMRU, OPPERF-DESCENTR, MOTIV-RECOMPENS, EDPERF-IMPACT, RESPMOTIV-COMLOC, PERFANGJ-ADM, OPPERF-RECOMP, FIN-EDPERF, MOTIV-DOTARE, EDMRU-MOBILIT, PROVOCMRU-LEGIS, RESPMOTIV-SAL, MOTIV-STATUT, OPPERF-MASSMED, PROVOCMRU-CONSULTCE, EDMRU-ASIGRES, PERFANGJ-SAL, EDMRU-BIROCR, BENMRU-PREDICT, FLEXMG-PERF, EDMRU-DEFPREG, PERFANGJ-PARINTI, MOTIV-SALARIZ, BENMRU-PERCEPSOC, PROVOCMRU-EXCELENTA, EDMRU-POLCOER, RESPMOTIV-PARINTI, MOTIV-STABIL, OPPERF-POLCOER, MOTIV-EDPERF, MOTIV-ORGANIZ, OPPERF-FORMCC, BENMRU-COLAB, PERFANGJ-ONG, BENMRU-COMPETITIE, PERFANGJ-EDMGSUP, EDMRU-MENTPERSCOMP, OPPERF-STATUT, RESPMOTIV-EDMGSUP, PERFANGJ-COMLOC, BENMRU-IMPLIC, EDMRU-MOTIV, RESPMOTIV-ADM, EDMRU-FORMCC.

The value of the non-standard Beta coefficients of the regression equation reveals that there is an inversely proportional variation for certain regression variables, such as inversely proportional dynamics of the aggregation of the solution complex in relation to the combination of school activity with extracurricular activities; decentralization of the education system in relation to the option to combine school activity with extracurricular activities in the context of performance; reducing bureaucracy in relation to increasing performance; improving the economic and social environment from which the student comes (national economic and social growth and the stimulation of partnerships with the authorities capable of effectively helping families from disadvantaged social backgrounds for the student).

**Figure 4:**
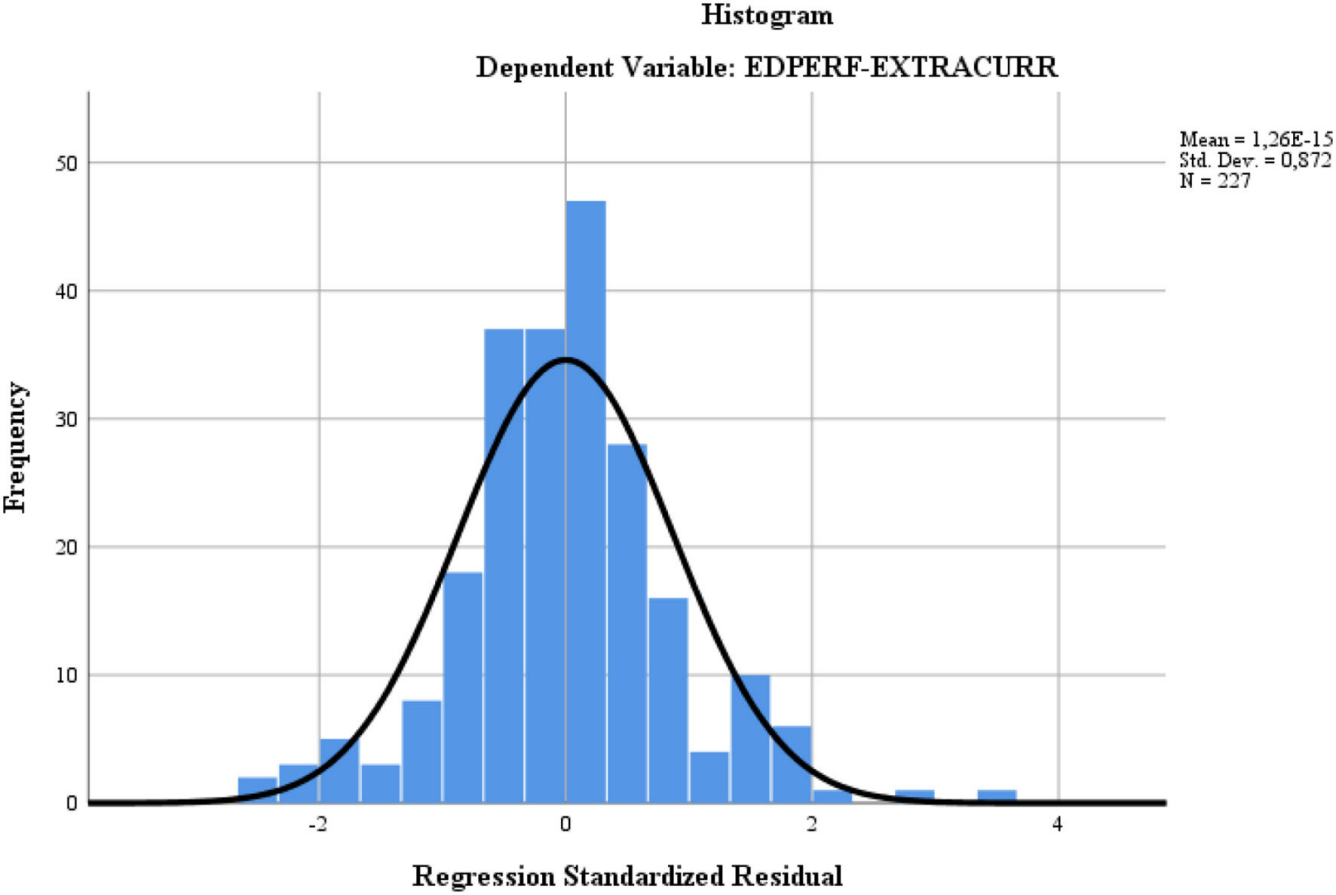
Histogram distribution of the motivational model.

It was found, through the histogram distribution (Figure 4), that the evolution of the dependent variables under the Gaussian curve is an optimal one with an inflection point at the maximum of the curve. From the dispersion of the results point of view, related to the integrated character of the model, we found that the countries closest to the trend curve are Spain, the UK, and Cyprus, the others, including Romania, summing up significant distances from the trend curve.

Another model aimed at comparing the set of regressive variables with motivation as a determinant of management policies. In this case, there was a statistically significant evolution of the model for a coefficient of determination of 72.5%, achieving the objective of validating the model through statistical tests for multiple linear regression (see Table 4). The model's dependent variable was established concerning the research objectives as the level of motivation of the teachers engaged in the educational process. The regressors are those presented above (in the previous model).

**Table 4:** Summary of the linear regression model on motivation as a determinant of the management policies.

**Model^a^^,^ ^b^**	***R***	***R* square**	**Adjusted *R* square**	**Std. error of the estimate**	**Change statistics**
					***R* Square change**	***F* Change**
Management policies	0.851	0.725	0.638	0.813	0.725	8.392
	**df1**		**df2**	**Sig**. ***F*** **Change**	**Durbin Watson**	
			54	172	0.000	1.742

a Predictors: (Constant), OPPERF-FORMCC, MOTIV-DOTARE, PROVOCMRU-CONSULTCE, MOTIV-CLIMAT, FIN-EDPERF, PERFANGJ-ELEV, OPPERF-DESCENTR, RESPMOTIV-ADM, EDMRU-DESCENTR, EDMRU-ANGPERSCOMP, EDMRU-MEDEC, EDPERF-EXTRACURR, MOTIV-ECHMRU, PROVOCMRU-EXCELENTA, MOTIV-RECOMPENS, PROVOCMRU-TRANSPCE, PERFANGJ-ADM, ANGPERSCOMP-EDPERF, PERFANGJ-EDMGINF, RESPMOTIV-SAL, MOTIV-STATUT, OPPERF-MASSMED, EDMRU-MOBILIT, EDMRU-ASIGRES, PROVOCMRU-LEGIS, BENMRU-PREDICT, EDMRU-BIROCR, FLEXMG-PERF, PERFANGJ-PARINTI, PERFANGJ-SAL, EDMRU-DEFPREG, MOTIV-SALARIZ, RESPMOTIV-EDMGINF, BENMRU-PERCEPSOC, RESPMOTIV-PARINTI, EDMRU-POLCOER, PROVOCMRU-RECOMPENS, OPPERF-RECOMP, MOTIV-STABIL, MOTIV-EDPERF, OPPERF-POLCOER, MOTIV-ORGANIZ, BENMRU-COLAB, PERFANGJ-ONG, BENMRU-COMPETITIE, PERFANGJ-EDMGSUP, EDMRU-MENTPERSCOMP, OPPERF-STATUT, EDPERF-IMPACT, BENMRU-IMPLIC, PERFANGJ-COMLOC, EDMRU-MOTIV, EDMRU-FORMCC, RESPMOTIV-COMLOC.

b Dependent Variable: RESPMOTIV-EDMGSUP.

The ANOVA test validated the model, the sum of the squares of the regressors being approximately three times higher than the sum of the residues for 54 degrees of freedom compared to 172 in the case of the residual variable (see Table 5).

**Table 5:** ANOVA table regarding motivation as a determining factor of the management policies.

**Model^a^^,^ ^b^**		**Sum of squares**	**d*f***	**Mean square**	***F***	**Sig**.
Management policies	Regression	299.517	54	5.547	8.392	0.000
	Residual	113.681	172	0.661		
	Total	413.198	226			

a Dependent Variable: RESPMOTIV-EDMGSUP.

b Predictors: (Constant), OPPERF-FORMCC, MOTIV-DOTARE, PROVOCMRU-CONSULTCE, MOTIV-CLIMAT, FIN-EDPERF, PERFANGJ-ELEV, OPPERF-DESCENTR, RESPMOTIV-ADM, EDMRU-DESCENTR, EDMRU-ANGPERSCOMP, EDMRU-MEDEC, EDPERF-EXTRACURR, MOTIV-ECHMRU, PROVOCMRU-EXCELENTA, MOTIV-RECOMPENS, PROVOCMRU-TRANSPCE, PERFANGJ-ADM, ANGPERSCOMP-EDPERF, PERFANGJ-EDMGINF, RESPMOTIV-SAL, MOTIV-STATUT, OPPERF-MASSMED, EDMRU-MOBILIT, EDMRU-ASIGRES, PROVOCMRU-LEGIS, BENMRU-PREDICT, EDMRU-BIROCR, FLEXMG-PERF, PERFANGJ-PARINTI, PERFANGJ-SAL, EDMRU-DEFPREG, MOTIV-SALARIZ, RESPMOTIV-EDMGINF, BENMRU-PERCEPSOC, RESPMOTIV-PARINTI, EDMRU-POLCOER, PROVOCMRU-RECOMPENS, OPPERF-RECOMP, MOTIV-STABIL, MOTIV-EDPERF, OPPERF-POLCOER, MOTIV-ORGANIZ, BENMRU-COLAB, PERFANGJ-ONG, BENMRU-COMPETITIE, PERFANGJ-EDMGSUP, EDMRU-MENTPERSCOMP, OPPERF-STATUT, EDPERF-IMPACT, BENMRU-IMPLIC, PERFANGJ-COMLOC, EDMRU-MOTIV, EDMRU-FORMCC, RESPMOTIV-COMLOC.

As in the case of the model focused on the active combination mode as an element of motivation (professional direction of HRM strategy), it was observed that the model based on the motivated self-interest of staff fit optimally under the Gaussian curve. The countries which adhere to motivational practices as HRM policies promoted at the level of the education system were Romania, Greece, Turkey, and Cyprus.

## Discussion

To achieve the research objectives, we used statistical query techniques of the general database for structural indicators in education and found that, in Romania, between 1991 and 2019, the annual average school population was around 4,320,000 enrolled in about 17,400 (average) educational institutions. The personnel policy involved an average of 300,000 teachers who ensured the fulfillment of the educational objectives assumed through the curricular documents and the national education strategies. During this period, significant changes were registered in the school population structure due to factors such as school dropout, urbanization, lack of qualified staff in rural areas, frequent changes in staff policy and curriculum, etc.

An analysis of the statistical data published by the National Institute of Statistics showed a significant reduction (400%) of educational units in the Romanian educational system during 1991–2019, from 28,303 units in 1991 to 7,020 units in 2019. These units experienced a structural change according to the age of those participating in education. For instance, in preschool education, the dynamics of the number of educational units has been constantly decreasing and its share reduced from 44% in 1991 to 17% in 2019.

**Figure 5:**
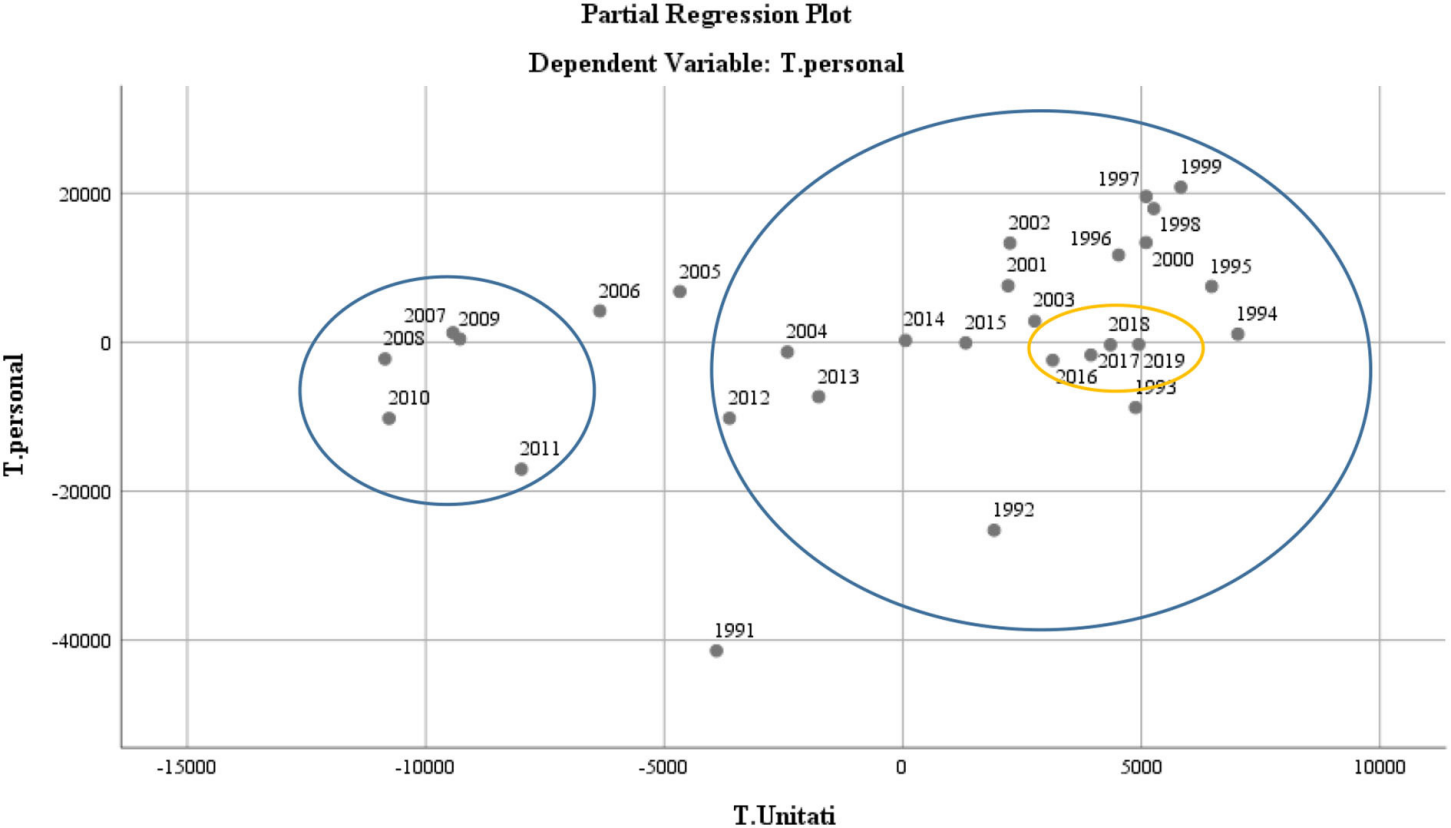
Scatter diagram representing the dynamics of the staff in the Romanian educational system with the dynamics of school units.

The analysis of the statistical data also showed a two-dimensional distribution in the dynamics of the number of school units with a positive increase in the post-December 1989 to 2003 period, and maintenance of the status quo in the 2018–2019 period (see Figure 5).

In the primary cycle, the number of units remained relatively constant as a share in the total number of profile units with a slight increase toward the end of the period above 50%, due to the restriction of the number of state units in pre-university education on preschool and vocational segments.

If in preschool education, the educational offer was taken over by private units; in vocational education, this contributed to the drastic reduction of representativeness (which since 2011 has reached 0% of the total existing educational institutions at the national level).

The lack of these institutions and the deprofessionalization of the students graduating from pre-university studies generated high economic tensions because of the overcrowding of skilled labor in some fields. The economic agents had to take over some assets of apprenticeship and training of qualified technical staff. In this context, there was a small recovery of vocational training units toward the end of the period in 2019, which brought their share in total units to 1%.

If the dynamics of educational units provided a material aspect of the demand for human resources in the pre-university education system by direct reference to the dynamics of the units, we could consider that the demand for human resources was decreasing, registering a decline in education. This aspect is false because, against the background of the development of the school curriculum and the curricular changes, the Romanian education system has been flexed in permanent expansion in recent years due to the European objectives of operationalization and efficiency of the education system, corroborated with the EU cohesion and development policies.

**Figure 6:**
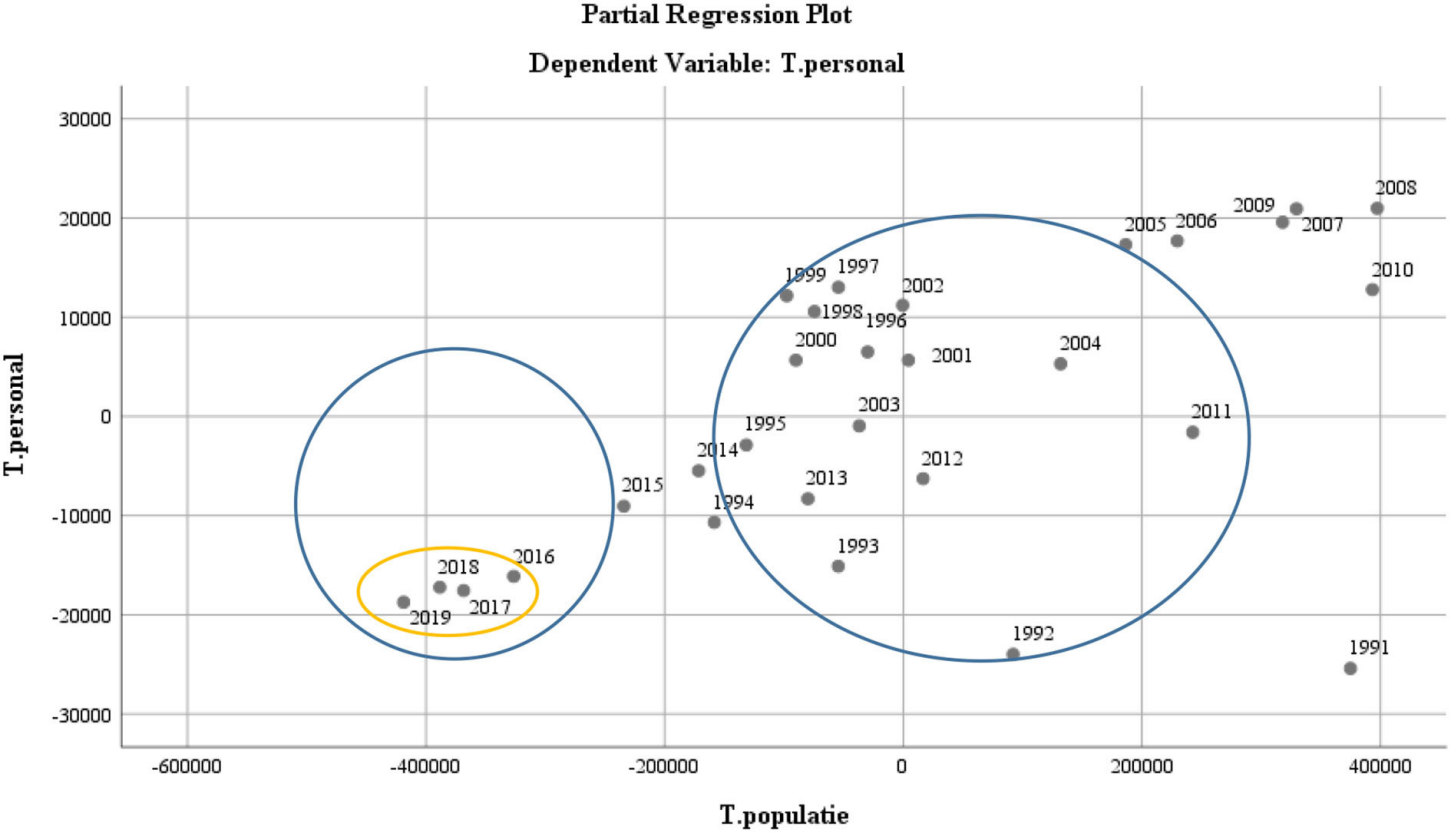
Scatter diagram—to represent the dynamics of the staff in the Romanian educational system with the dynamics of the school population.

It is interesting to study the school population and its structure represented horizontally (over time) and vertically, by age levels related to the various stages of student training. During 1991–2019, the dynamics of the school population increased against the background of the access to the system of the preparatory class and the increasing accessibility of education through digitalization, which required the valorization of human resources about the needs of the school population, the efficiency of the human resource in the education system based on the educational objectives, and the development policies of the education promoted in Romania (see Figure 6).

From the data provided by the National Institute of Statistics, it was observed that the preschool population maintained its relatively constant level of representation in the total school population at 15%, the school population in the gymnasium cycle decreased by at least 7% at the end of the period compared to the beginning of 1991, while the population in the vocational education system had a representation of 0% in 2011. Subsequently, until the end of the period, there was (under the impact of pressure from economic agents) a small recovery that brought it to a percentage of representativeness of 3%. This meant that pre-university vocational training was not very desirable in Romania, with the same situation persisting for the school population employed in post-secondary education.

The dynamics of the personnel in the pre-university education system is, in the context of those previously analyzed, increasing up to three times during the analyzed period. The most significant share was found in the primary and secondary education system in which about 50% of the teachers in the education system were active at the end of the period.

In the second place, the personnel employed in high schools show an ascending trend (of 4% during the analyzed interval), and in the third place, the personnel from preschool education. The staff from vocational or post-secondary schools were very poorly represented (critical level 0 being reached in 2011) because, currently, the level of representativeness of these two categories is 1%.

**Figure 7:**
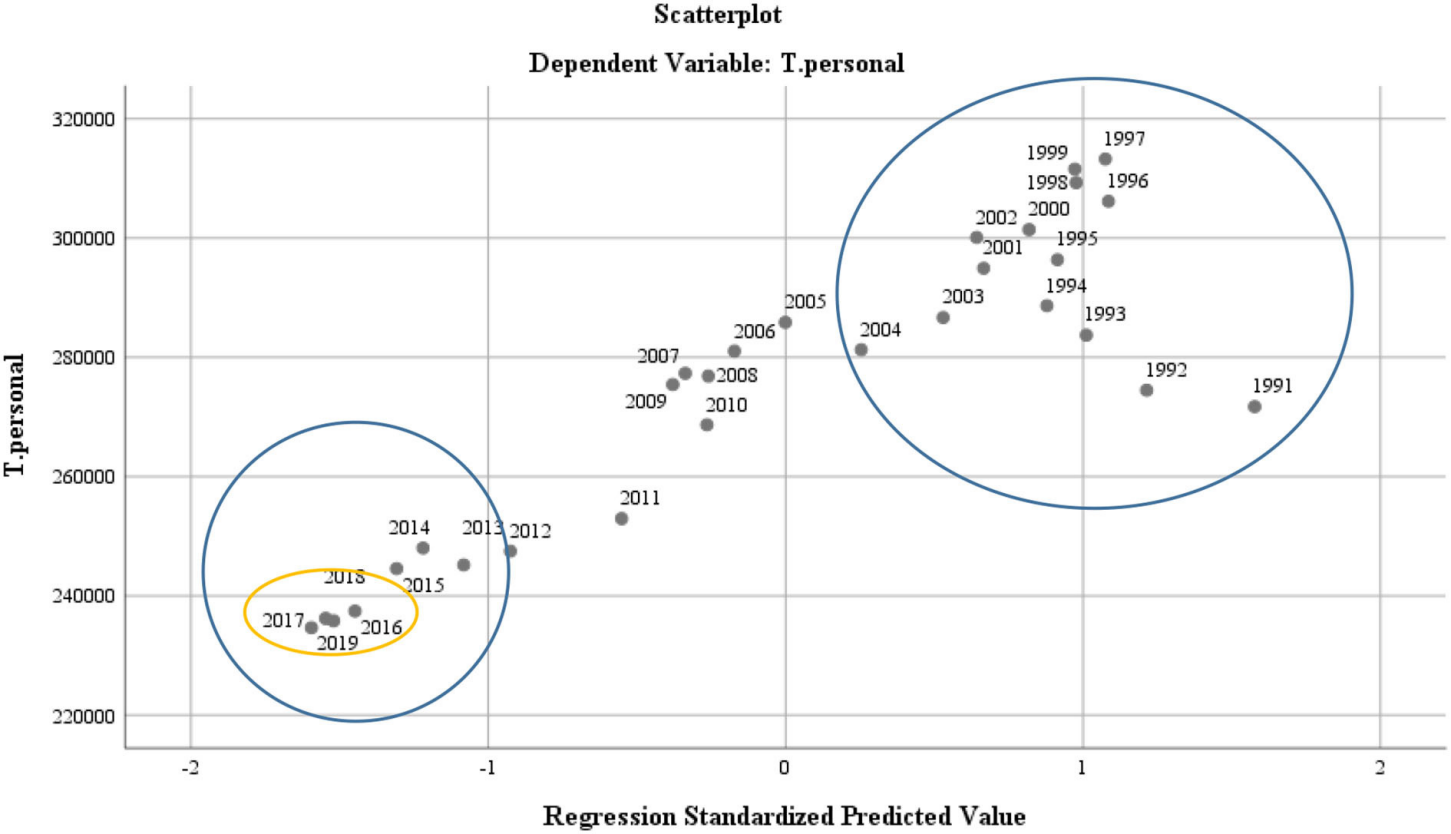
Scatter diagram—for representing the dynamics of the staff in the Romanian educational system.

At the level of personnel policy dynamics, a two-dimensional distribution with positive representation in the period 1989–2003 and a contraction in the current period 2018–2019 was observed (see Figure 7).

The above analysis demonstrates that the structuring based on curricular documents of the learning contents was the main factor generating the demand for human resources in the Romanian education system. It focused in particular on the basic training levels based on the minimum knowledge of the student and was characterized by a poor representation of performance at the transdisciplinary and inter-curricular levels.

***Hypothesis 1*** on *Performance in education is the result of combining school activity with extracurricular activities if and only if the teacher's perception of organizing extracurricular activity is directed to activities with concrete results and positive impact on performance* was validated based on the statistical results from the modeling, which was designed based on questionnaire responses relating to the dependent variable: “Performance in education represents the result of combining school activity with extracurricular activities” (EDPERF-EXTRACURR). The model's statistical representativeness, which validated the working hypothesis, was over 70%.

***Hypothesis 2*** on *Teachers can be motivated by HRM actions/strategies aimed mainly at promoting a correct status of teachers, attracting the local community and educational partners in collaboration with teachers* was demonstrated by both proposed models—the model that captured the correlation between intrinsic and extrinsic motivating factors, and the model quantifying the role of HRM in the process of obtaining school performance knowing the quantification of motivation as a factor generating performance.

In the case of ***hypothesis 3*** on *Increasing flexibility and creating adequate tools to facilitate bureaucratic tasks will have a direct effect on teacher performance*, the modeling premises ensured its validation with the rejection of the null hypotheses for proposed econometric models. The aspects regarding the use of appropriate tools were captured in the regression variables, which addressed the administrative aspects of HRM and the performance evaluation aspects.

The Pearson's correlation table compared the interest of the senior management with that of the middle management. The results revealed that the correlation coefficients on the dependent variables of the models presented superior values when using the instruments of interest at the level of senior management compared to the values for middle-level management. This aspect validated ***hypothesis 4*** on *The interest of the high-level management in providing staff motivation tools is one of the desirable strategies with an impact on teachers*.

## Conclusions

The proposed models manage to capture the differences between the theoretical and practical approaches to the educational development strategy based on skills and performance, building a useful tool for decision-makers to adjust vulnerabilities through concrete measures such as combining school activity with extracurricular activities and motivating the teaching staff through HRM actions/strategies. These strategies are mainly aimed at promoting the status of teachers by attracting the local community and the educational partners in collaboration with the teachers, increasing flexibility and creating appropriate tools to facilitate bureaucratic tasks, and involving high-level management in providing motivational tools to staff.

The findings of this current research suggest that there is a relationship of direct proportionality and dependence between the vectors of the school activity (i.e., number of school units, school population, and staff employed in the education system). It follows that the intrinsic motivation for the efficiency and effectiveness of the human resource management process in the education system are the educational goals getting translated into clearly defined training objectives (SMART type) that motivate the human resources professionally as a factor of competitiveness and access to the system.

The limits of this approach are in the absence of the pecuniary character of the offer, an aspect that is likely to significantly influence the performance of the human resource and the selected management strategies for the operationalization of this resource.

## Data availability statement

The original contributions presented in the study are included in the article/supplementary material, further inquiries can be directed to the corresponding author/s.

## Ethics statement

Ethical review and approval was not required for the study on human participants in accordance with the local legislation and institutional requirements. Written informed consent from the patients/participants or patients/participants legal guardian/next of kin was not required to participate in this study in accordance with the national legislation and the institutional requirements.

## Author contributions

Conceptualization and writing—original draft preparation: RI, VA, and MZ. Methodology: RI, VA, MR, and MZ. Validation: MZ, VA, MR, and NC. Formal analysis: VA, NC, and MZ. Investigation: NC, MR, and RI. Resources: MR, NC, and RI. Writing—review and editing: MR and NC. All authors have read and agreed to the published version of the manuscript.

## Conflict of interest

The authors declare that the research was conducted in the absence of any commercial or financial relationships that could be construed as a potential conflict of interest.

## Publisher's note

All claims expressed in this article are solely those of the authors and do not necessarily represent those of their affiliated organizations, or those of the publisher, the editors and the reviewers. Any product that may be evaluated in this article, or claim that may be made by its manufacturer, is not guaranteed or endorsed by the publisher.
